# Mast Cells May Differentially Regulate Growth of Lymphoid Neoplasms by Opposite Modulation of Histamine Receptors

**DOI:** 10.3389/fonc.2019.01280

**Published:** 2019-11-21

**Authors:** Sandeep Paudel, Deeksha Mehtani, Niti Puri

**Affiliations:** Cellular and Molecular Immunology Lab, School of Life Sciences, Jawaharlal Nehru University, New Delhi, India

**Keywords:** mast cells, mast cell mediators, tumor cells, lymphoid neoplasms, histamine receptors, apoptosis

## Abstract

Cancer microenvironment is complex and consists of various immune cells. There is evidence for mast cell (MC) infiltration of tumors, but their role thereof is poorly understood. In this study, we explored the effects of mast cell and their mediators on the growth of hematological cancer cells. The affect is demonstrated using RBL-2H3 MCs, and YAC-1, EL4 and L1210 as hematological cancer cell lines. Direct contact with MCs or stimulation by their mediators caused growth inhibition of YAC-1 cells, growth enhancement of EL4 cells and no change in growth of L1210 cells. This effect was confirmed by cancer cell recovery, cell viability, mitochondrial health, and cell cycle analysis. MCs showed mediator release in direct contact with tumor cells. MC mediators' treatment to YAC-1 and EL4 yielded exactly opposite modulations of survival markers, *Survivin and COX-2* and apoptosis markers, Caspase-3, Bcl-2, in the two cell lines. Histamine being an important MC mediator, effect of histamine on cell recovery, survival markers and expression of various histamine receptors and their modulation in cancer cells was studied. Again, YAC-1 and EL4 cells showed contrary histamine receptor expression modulation in response to MC mediators. Histamine receptor antagonist co-treatment with MC mediators to the cancer cells suggested a major involvement of H2 and H4 receptor in growth inhibition in YAC-1 cells, and contribution of H1, H2, and H4 receptors in cell growth enhancement in EL4 cells. L1210 showed changes in the histamine receptors' expression but no effect on treatment with receptor antagonists. It can be concluded that anti-cancerous action of MCs or their mediators may include direct growth inhibition, but their role may differ depending on the tumor.

## Introduction

Cancer can be characterized by indefinite multiplication, promotion of metastasis, and invasion by evading growth suppressors, defying apoptosis, invigorating angiogenesis, signaling to proliferate constantly, evade immune destruction, exclusion of cell energy limitation, instability in the genome and multiple mutations, and enhanced inflammation in tumors (1). Cancer microenvironment is complex and comprises various types of cells like endothelial cells, fibroblast, and immune cells. It also consists of cytokines, extracellular matrix (ECM), growth factors etc. surrounding the tumor cells. Solid tumors are complex heterogeneous structures comprising cancer and stromal cells embedded in an ECM and fed by the vascular network. The tumor tissue is linked with an altered ECM and fibroblasts which synthesize growth factors, chemokines, and adhesion molecules. Whereas, bone marrow makes the tumor microenvironment for hematologic malignancies, with blood vessels and peripheral lymphoid organs that provide cytokines and growth factors for tumor growth and survival (2). Along with this some soluble factors like VEGF and G-CSF and many others are present that maintain the growth and survival of hematologic tumors. Cancer cells in the solid tumors and hematological malignancies receive survival signals from the tumor microenvironment and it is therefore an important factor affecting the cancer treatments (3).

It is established that immune cells infiltrate cancer microenvironment with much focus on the T-lymphocytes, natural killer (NK) cells, and dendritic cells (4). MCs have also been found to infiltrate tumors and the role played by them in the tumor microenvironment is favorable or damaging to tumors dependent on tumor location, tumor stage, and chemotactic agents in the microenvironment (5). MCs are well-known as effectors of allergy and inflammation. MCs are spread in various tissues all over the body, but a substantial number of them are situated near blood vessels, nerves, and mucosal surfaces, and prominently present in the dermis, hypodermis, respiratory, and gastrointestinal tract (6). MC phenotype and maturation, are influenced by the local microenvironment and thereby their response to various stimuli by releasing an array of biologically active mediators. These characteristics make MCs as first responders in threats and also respond to changes in their environment by communicating with a variety of other cells implicated in physiological and immunological responses. From past two decades, MCs have been acknowledged due to their participation in many physiological and pathological processes. The most important feature of MC biology is the presence of numerous secretory granules occupying the major proportion of the cytoplasm of these cells (7). MCs activation can bring about release of preformed mediators that are stored in its cytoplasmic granules like biogenic amines (histamine, serotonin), lysosomal enzymes (cathepsin B, cathepsin E, β-hexosaminidase), cytokines, and growth factors [tumor necrosis factor (TNFα), IL-4, VEGF]; neoformed lipid mediators that are membrane lipid derived, like phospholipids (Prostaglandin D2, Leukotrienes); and neosynthesized mediators that are produced on transcriptional activation, like cytokines (IL-33, IL-10, IL-12, TNFα), growth factors (GM-CSF, TGF-β, VEGF) (8). The release of these MC mediators lead to a physiological or pathophysiological event (9). Therefore, role of MCs is critical in both infectious diseases like leishmaniasis, tuberculosis, chagas disease (10), and non-infectious diseases like atherosclerosis, asthma, arthritis, allergies (11) and also in cancer (12–14). Hence to study the role of MCs in cancers is intriguing and is of great importance.

MCs are attracted toward the tumor cells and recruited to tumor microenvironment by chemotactic molecules expressed by tumor cells themselves (15). MCs are the important source of pro-angiogenic factors and also influence the progression of cancer by modulating the cancer microenvironment by producing matrix metalloproteinases (MMP-2, MMP-9), and proteases (tryptase and chymase) that play a central role in ECM degradation and allow cancer cells to invade the microenvironment and metastasize to diverse locations (16). MCs not only play a role in solid cancer but also in hematological malignancies like lymphomas and leukemias. MCs have been shown to increase angiogenesis in canine lymph node lymphoma (17). Also MCs can be a good prognostic markers as their numbers are increased in skin biopsies of patients with cutaneous T cell lymphoma and cutaneous B cell lymphomas (18). It has been found that tumors which contains fibroblasts in the microenvironment, get affected by the heparin. In a study of breast cancer, head and neck cancer, lung cancer, ovarian cancer, and non-Hodgkin's lymphoma, it has been established that MCs release heparin which inhibits tumor growth in co-culture of cancer cells with fibroblasts (19). Out of all the mediators of MCs, Histamine is a mediator of prime importance (20, 21). Histamine is a pre-formed mediator present in the granules of MCs. Histamine exhibits anti-proliferative effects in the experimental models of human mammary cancer, melanoma, and cholangiocarcinoma (22). Histamine even potentiates the effects of radiation therapy, as seen in human breast cancer cells (22). Histamine is a growth factor capable of regulating cell proliferation via its histamine receptors (H1R, H2R, H3R, and H4R) (23–26). Histamine receptors differ in their expression patterns and functions and their activation mediates various biological effects (21). Increasing evidences imply that MCs gather along the tumors and could promote or suppress the tumor growth, migration, invasion, metastasis depending on the stage, and localization of tumor.

MCs have been found to play contrasting roles in cancer but whether the role of these cells is favorable or damaging in hematological malignancies is still unclear. The present study is of great importance as nowadays there is much focus on learning about the immune cells and their protective roles in cancers. Therefore, we explored the mechanisms to increase our understanding about the interaction of MCs and hematological cancers. We found that MCs played differential roles in different hematological malignancies of lymphoid origin and further we looked into the receptors involved and their signaling. It is known that MCs can influence the function of regulatory cells of immune system therefore with our better understanding of the mechanisms and pathways involved in the interaction of cancer cells with MCs, we can further look into development of new therapeutics to target the receptors or molecules involved in the signaling that tackle the hematological malignancies with the help of MCs or their mediators.

## Materials and Methods

### Animals

To isolate splenocytes, inbred C57BL/6, or SWISS mice (8–12 weeks age, 20–25 g) were used. The animals were safely housed in Central Laboratory Animal Resource Facility at JNU, New Delhi or obtained from the National Institute of Nutrition, Hyderabad, India. Positive-pressure was maintained in the animal house and temperature at 25°C, relative humidity at 50%. The animals were kept on a 12 h light/dark cycle maintaining pathogen free conditions. Water and mouse chow were provided *ad libitum*. Approval for the experimental protocols was taken from Institutional Animal Ethics Committee (IAEC) JNU, New Delhi (registration no: 19/GO/ReBi/S/99/CPCSEA) (IAEC: 13/2013). Every experiment was conducted under relevant guideline and regulations.

### Isolation and Culture of Mouse Splenocytes

Spleen cells were obtained from 8 to 12week old C57BL/6 or SWISS mice using the method previously described by Alam et al. (27). Briefly, spleen was isolated from mouse then gently minced in PBS (Phosphate Buffered Saline). The cell suspension was passed through nylon mesh and centrifuged at 391 g at room temperature to obtain cell pellet. Erythrocytes were lysed using sterile double distilled water. Double distilled water was then neutralized by adding the same volume of 2X PBS. Suspension was again centrifuged at 391 g at room temperature and the cell pellet obtained was resuspended in Roswell Park Memorial Institute-1640 medium (RPMI-1640) media (Sigma Aldrich) containing 10% FBS (Gibco, Life technologies, Grand Island, NY, USA) and β-mercaptoethanol (Sigma Aldrich). The cells were counted in a haemocytometer and then used for the experiments.

### Maintenance of Cell Lines

Murine T lymphocyte (EL4) and Murine lymphoblast (YAC-1) cell lines were obtained from (ATCC, MD, USA). Lymphocytic B cell (L1210) and B cell hybridoma (TIB-142) cells were obtained from National Center for Cell Sciences (NCCS), Pune, India. RBL-2H3 was a kind gift from Dr. Paul Roche, NIH, USA. The cell lines, EL4 and TIB-142, were cultured in Dulbecco's Modified Eagle's Medium (DMEM) media (Sigma Aldrich) and Murine lymphoblast; YAC-1 cells were cultured in RPMI media (Sigma Aldrich). Media were supplemented with 300 μg/ml glutamine, 20 mM HEPES (Sigma, MO, USA), 2 × 10^−5^ M β-Mercaptoethanol, 40 μg/ml gentamicin and 10% heat inactivated Fetal Bovine Serum (FBS) (Gibco, Life technologies, Grand Island, NY, USA). Rat Basophilic Leukemia (RBL-2H3) mast cell line was cultured in media containing equal proportion of Iscove's Modified Dulbecco's Medium (Gibco, Life technologies, Grand Island, NY, USA) and Minimum Essential Medium Eagle with Earle's salts (Gibco, Life technologies, Grand Island, NY, USA) media supplemented with 300 μg/ml glutamine, 20 mM HEPES, 2 × 10^−5^ M, 2-Mercaptoethanol, 40 μg/ml gentamicin and 20% heat inactivated Fetal Bovine Serum (FBS) (28). All the cells were maintained at 37°C in humidified atmosphere containing 5% CO_2_ (29). EL4, TIB-142, and YAC-1 cells were maintained as suspension cultures and subcultured by harvesting using centrifugation whereas RBL-2H3 cells were maintained as adherent culture and subcultured by trypsinization and further harvested using centrifugation. Description of various cell lines used is given in Supplementary Table 1.

### Stimulation of RBL Mast Cells (MCs) and Generation of Mediators

Mast cell (RBL-2H3) exocytosis was induced by 2,4-Dinitrophenylated Bovine Serum Albumin, DNP-BSA (was a kind gift from Dr. Paul A. Roche, NCI, NIH, USA), as described earlier (30). Briefly 0.3 × 10^6^ cells/well were seeded in 24 well plates and sensitized with DNP-specific IgE (TIB-142 hybridoma culture supernatant) and then cross-linked with 100 ng/ml DNP-BSA. Supernatants and lysates were collected after 45 min of exocytosis induction. The release of β-hexosaminidase was determined by using an enzymatic assay as described (31). For the experiments, mediators were collected from RPMI (completely resting, where MCs were treated only with RPMI), Resting MC (MC not sensitized with IgE but treated with 100 ng/ml DNP-BSA), Sensitized MCs (MC treated with IgE) and activated MCs (MC were sensitized with IgE and further cross-linked with 100 ng/ml of DNP-BSA).

### Preparation of Tumor Cell Conditioned Supernatant and Stimulation of RBL-mast Cells

0.15 × 10^6^ tumor cells/well in 48 well plates were seeded in RPMI PR^−^ media and incubated for 6, 12, and 24 h. Tumor cell conditioned supernatants were collected at 6, 12, and 24 h (32). The supernatants were then used to stimulate 0.15 × 10^6^ RBL cells/well seeded in 48 well plates for 6, 12, and 24 h. The resting cells were treated with RPMI PR^−^ media incubated at 37°C for 6, 12, and 24 h in the same well plate. Supernatants and lysates were collected from resting and tumor cell supernatant treated RBL mast cells. As a positive control, RBL MCs were activated with DNP-BSA using the protocol described above in section Stimulation of RBL Mast Cells (MCs) and Generation of Mediators. The release of β-hexosaminidase was determined by using an enzymatic assay as described (31).

### Co-culture of MCs and Tumor Cells *in vitro*

0.15 × 10^6^ MC alone and along with tumor cells were cultured for 6, 12, 24 h in 48 well plate, supernatants and lysates were collected and release of β-hexosaminidase was determined by using an enzymatic assay as described (31). 0.09 × 10^6^ MC, tumor cells and MCs along with tumor cells were cultured in 48 well plate for 48 h for the flow cytometry experiment. YAC-1 + RBL were co-cultured in RPMI complete media and EL4 + RBL was co-cultured in DMEM complete media. To calculate the cell numbers at 48 h for the co-culture, total cells were counted and then according to the percentage of the stained cells in the co-culture obtained by flow cytometry, the number of tumor cells and RBL cells were calculated. To calculate the number of RBL cells in the coculture, the formula used is total number of cells counted × percentage of stained cells (in the coculture) and divided by 100. Similarly to calculate the number of tumor cells in the coculture, the formula used is total number of cells counted × percentage of unstained cells (in the coculture) and divided by 100. For the MTT assay, 0.01 × 10^6^ of both MC and tumor cells were co-cultured for 0, 12, 24, and 48 h with MC mediators in 96 flat bottom well plate and growth was assessed. For the transwell co-culture experiments, in the lower chamber of transwell 0.3 × 10^6^ tumor cells were seeded [Polycarbonate membrane with 0.4-μm pore size, 6.5-mm diameter (Corning Costar, Cambridge, MA)] and 0.3 × 10^6^ MCs were added to the top chamber of the transwell plate in 24 well plates. Later the viability of MCs and tumor cells was evaluated through trypan blue dye exclusion assay.

### Detection of Cell Metabolic Activity by MTT (3-(4,5-Dimethylthiazol-2-yl)-2,5-Diphenyltetrazolium Bromide) Assay

Cell proliferation and metabolic activity was assessed by monitoring the conversion of MTT to formazan as described earlier by Naqvi et al. (28). Briefly, seeding of cells was carried out as 100 μl/well in 96 well-flat bottom microtiter plates. Tumor cells were treated with mediators prepared *in vitro* from activated or resting MCs or different concentrations of histamine (Sigma Aldrich) for specific time periods. 20 μl of filter sterilized MTT (5 mg/ml in PBS) was added at specific time points. After incubating 4 h with MTT, formazan crystals that were formed were dissolved in 100 μl sterile dimethyl sulfoxide (DMSO) followed by incubation at 37°C for 30 min. The absorbance was then measured at 595 nm with a Spectra Max M2 plate reader. The growth curve was plotted as absorbance (blanked with MTT+DMSO, without cells) against time. The experiment was performed in triplicates.

### Flowcytometric Analysis to Detect Cell Surface Receptor

Briefly, 0.2 × 10^6^ cells were suspended in staining buffer containing PBS along with 2% FBS and 0.09% Sodium azide. Before staining, cells were incubated on ice for 20 min with anti-mouse CD16/32 Fc block (1 μg for 1 × 10^6^ cells) (Biolegand, San Diego, CA, USA). Incubation was carried out with mouse anti IgE- FITC or mouse anti IgE- PE (Biolegand, San Diego, CA, USA) and also with their isotype controls for 30 min on ice. After staining, washing was done twice with PBS and cells were immediately analyzed in flow cytometer. Ten thousand cells were examined on BD FACS calibur by using Cell Quest Software. The percentage calculation shown in the result was obtained by dividing IgE-positive cells with total cells and multiplying by 100.

### Detection of Apoptosis and Necrosis

Briefly, 0.1 × 10^6^ cells were pre-treated with activated or resting MC supernatants for specific time periods. Staining was done using the method earlier described (28). Briefly, treated cells were stained with two stains i.e., Fluorescein isothiocyanate (FITC) conjugated Annexin V (Biolegand, San Diego, CA, USA) and 7-Aminoactinomycin D (7AAD) (Biolegand, San Diego, CA, USA) then washed with annexin binding buffer. Ten thousand cells were analyzed by cell quest software using Flow cytometry BD FACS Calibur.

### Cell Cycle Analysis

The effect of resting or activated MC supernatant on cell cycle was determined by flow cytometry with propidium iodide PI (Sigma Aldrich) staining of cells as described earlier (29). Briefly, 0.1 × 10^6^ cells were pre-treated with mediators from activated or resting MCs for 0, 12, 24 h. Cells were washed and then fixation was done with 70% ethanol overnight at 4°C. Treatment of fixed cells with 80 μg/mL RNase A (Sigma Aldrich) and 50 μg/mL PI in saponin-EDTA at 37°C for 30 min was carried out. Ten thousand events were acquired by cell quest software using Flow cytometry BD FACS Calibur and analyzed using MOD FIT software after appropriate gating, to determine the percentage of cells in each phase of the cell cycle.

### Estimation of Mitochondrial Membrane Potential

The mitochondrial membrane potential (Δψ) of cells was measured using Mitochondrial Membrane Potential Detection Kit (Invitrogen) (33). Briefly 0.1 × 10^6^ cells were pre-treated with activated or resting MC supernatants for 12, 24 h. Cells pellet was washed twice with PBS and re-suspended in 5 mM JC-1 and incubated for 30 min at 37°C in dark. Fluorescence emission was analyzed for 10,000 cells by flow cytometry (JC-1 monomers: excitation wavelength 488nm, emission filter 530/30nm; JC-1 aggregates: excitation wavelength 488nm, emission filter 585/42nm) using Cell Quest Software in BD FACS Calibur (Becton Dickinson, USA).

### Cell Viability Assay

Cells were pre-treated with activated or resting MC supernatants or Histamine (10 μM) (Sigma Aldrich) and/or Histamine receptor antagonist Pyrilamine (10 μM) (Sigma Aldrich), Ranitidine (10 μM) (Sigma Aldrich), and JNJ7777120 (10 μM) (Sigma Aldrich) in complete medium for specific time periods. Viable cell number was counted by trypan blue dye exclusion assay and hemocytometer.

### Primer Designing

*Mus musculus* specific primers were designed for *GAPDH, Histamine Receptor1, Histamine Receptor2, Histamine Receptor3, Histamine Receptor4, Survivin, COX-2* [prostaglandin-endoperoxide synthase 2 (Ptg2)], genes using Primer-BLAST software by setting the parameters as: 150–500 bp amplicon size for reverse transcription PCR, 20–24 base long primer, GC content of 50–55% with no self-complementarity. OligoCalc and Multalign were used to further validate the primers designed. Primer pairs used for PCR amplification are listed in the Supplementary Table 2.

### Isolation of RNA and First Strand (cDNA) Synthesis

Trizol chloroform method was used to extract RNA from cells as described earlier (34). Briefly, Trizol-chloroform was added to 5 × 10^6^ cells and then isopropyl alcohol was used to pellet down RNA from the aqueous layer. Seventy-five percent ethanol was used to wash the pellet twice. RNA pellet was dried and suspended in Tris/borate/EDTA (TBE) buffer. After confirming the purity and integrity, the RNA was used to prepare cDNA. M-MuLV RT enzyme (200 U/μl) (NEB) and Oligo (dT) 18 (50 μM) (NEB) in a reaction mixture of 15–20 μl was used to obtain cDNA which was used for the PCR amplification studies. For the quantitative RT PCR, we standardized the amount of RNA/reaction mix for the linear range.

### Semi Quantitative Reverse Transcriptase PCR

Amplification of cDNA obtained was done using gene specific primers designed and listed in Supplementary Table 2. Briefly, 1 μl of cDNA sample was used to prepare a 10 μl reaction mixture containing Taq Pol enzyme (5 U/μl) (NEB), dNTPs mix (0.25 mM) (NEB), Taq Pol enzyme buffer (1X) (NEB), forward and reverse primer (0.2 μM each), and Nuclease free water (Genei). The tubes were placed in Mastercycler pro (with vapo. protect, Eppendorf) for PCR amplification. The obtained amplified PCR products were run on 1.8% agarose gel after mixing with DNA loading dye. Gel electrophoresis was carried out at 90 V, bands visualized and the band intensity measured using Quantity One Software version 4.6.5 (Basic) from BIO-RAD. GAPDH was used as endogenous control.

### Immunoblotting

Cells were pretreated with activated or resting mast cell supernatants for 24 h. Cell lysate preparation was carried out as described (35). In brief, cells were lyzed using the lysis solution (50 mM Tris, 1% Triton X-100, 0.1% SDS, 150 mM NaCl) containing protease and phosphatase inhibitors (5 mM iodoacetamine, 50 mM PMSF, and 0.1 mM TLCK) (Thermo Scientific). The cell lysate was centrifuged at 20,854 g for 30 min. Protein were quantified using Bradford reagent and 40–60 μg protein was resolved on 12% SDS PAGE and transferred onto the polyvinylidene difluoride (PVDF) membrane. Blocking of membrane was carried out for 1 h by tris-buffered saline containing 0.05% Tween-20, and 5% (w/v) non-fat dry milk. Overnight incubation with primary antibody was carried out at 4°C. Primary antibodies used are anti-mouse PARP antibody (Cell Signaling Technology, Danvers, MA), anti-mouse Survivin antibody (Cell Signaling Technology, Danvers, MA), anti- mouse Caspase-3 antibody (Cell Signaling Technology, Danvers, MA), anti- mouse BCL-2 antibody (Cell Signaling Technology, Danvers, MA). After which incubation with secondary antibody was carried out, the immune-reactive bands were visualized using enhanced chemiluminescence method. The blots were then washed and re-probed with loading control, anti-β-actin antibody (Sigma).

### Statistical Analysis

Each experiment was repeated at least three times and data are plotted as mean ± SEM from data obtained from independent experiments. To test for significance of differences between different sets of data Non-paired Students *t*-test was done using Sigma plot software. Comparisons were considered significant at *p* < 0.05.

## Results

### Inhibition of Growth of YAC-1 Cells on Co-culture With RBL-MCs

**Figure 1:**
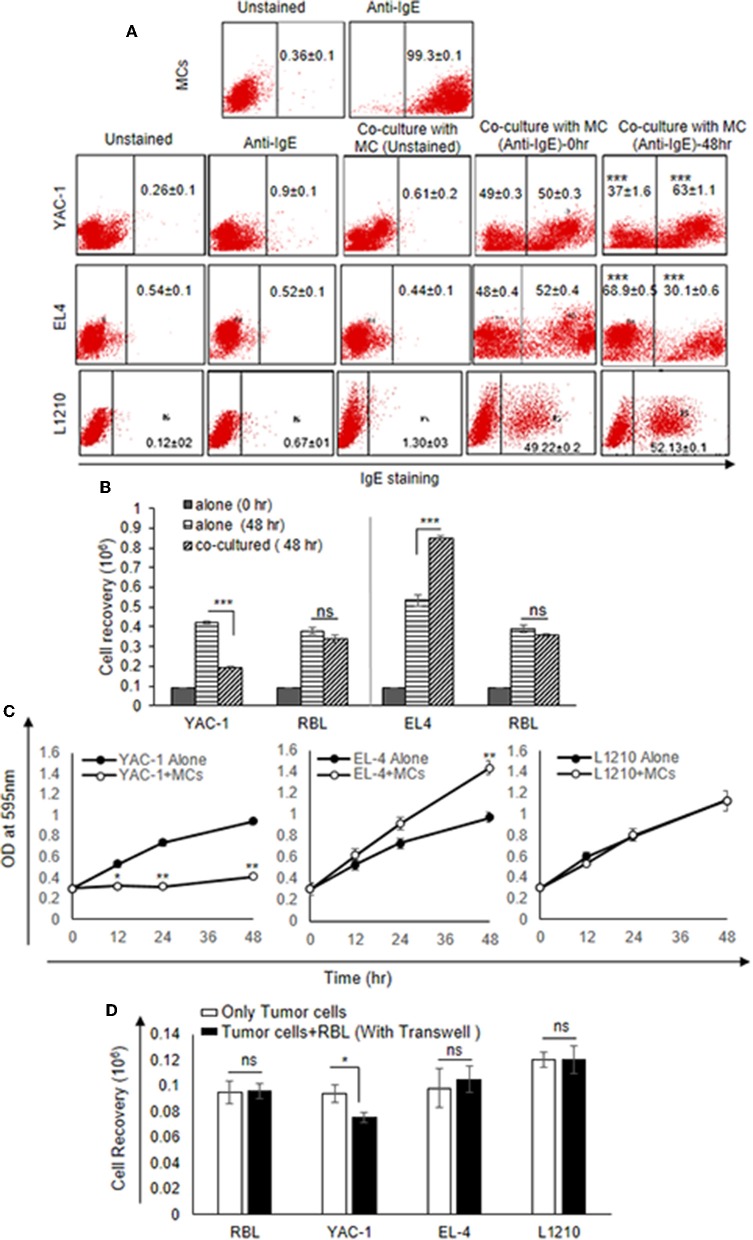
Direct Effects of MC on the growth of tumor cells. **(A)** Direct effect of MCs on tumor growth was assayed using flow cytometry. 0.09 × 10^6^ MCs, tumor cells and MCs with tumor cells were cultured in 48 well plate for 48 h and incubated with IgE (1:100) ratio for 3 h. Cells were harvested, washed, blocked by Fc block antibody and then stained with anti-IgE antibody (fluorescence conjugated) and analyzed on BD FACS Calibur using Cell Quest Software. **(B)** Effect of co-culture on the cell recovery of tumor cells and MCs. The tumor cells were counted after 48 h of incubation with MCs. The cell recovery of tumor cells and MCs, respectively, in the co-culture was calculated using the flow cytometry percentages as described in materials and methods. **(C)** 0.01 × 10^6^ both MC and tumor cells were co-cultured for 0, 12, 24, and 48 h with MC mediators in 96 flat bottom well plate and growth was assessed by MTT assay. **(D)** Co-culture experiment was assayed by transwell. 0.3 × 10^6^ tumor cells were cultured in lower chamber of a transwell polystyrene plate and 0.3 × 10^6^ MCs to the top chamber of the transwell plate in 24 well plates. Later the cell recovery of MCs as well as tumor cells was assessed through trypan blue dye exclusion assay. Data are expressed in as means ± SEM of three separate experiments, each sample was counted in duplicate. Data was analyzed using *t*-test ^*^p < 0.05, ^**^p < 0.005, ^***^P < 0.0005, ns, not significant.

Since MCs are an important component in tumor microenvironment (36) and there may be important crosstalk between MCs and tumor cells, the direct interaction between MCs and three murine tumor cells of hematological origin namely, two T cell lymphomas YAC-1 and EL4 and one leukemia L1210 was studied. Initially, the direct interaction of MCs with tumor cell in co-culture experiment was studied. RBL MCs were distinguished from tumor cells based on their surface expression of FcεRI receptor. They were co-cultured for 48 h and analyzed through flow cytometry (Figure 1A). Co-culturing MCs with YAC-1 cells resulted in a reduction of YAC-1 cell population from 49 ± 0.3 to 37 ± 1.6% and increase in MCs population from 50 ± 0.3 to 63.1 ± 1.15% with increase in time interval from 0 to 48 h. We found that YAC-1 cells alone have increased from 0.1 × 10^6^ to 0.42 × 10^6^ and RBL MCs cells alone have increased from 0.1 × 10^6^ to 0.38 × 10^6^ in 48 h (Figure 1B) whereas YAC-1 cells co-cultured with RBL MCs, calculating cell numbers at 48 h from the IgE staining percentages shown in the Figure 1A, YAC-1 cell numbers were found to be 0.19 × 10^6^ and RBL MC numbers were 0.34 × 10^6^ (Figure 1B). However, co-culturing RBL MCs with EL4 cell line resulted in an increase of EL4 cell population from 50 ± 0.4 to 68.9 ± 0.5% and a decrease in MCs population from 50 ± 0.4 to 30.1 ± 0.6%, with increase in time interval from 0 to 48 h. We found that EL4 cells alone have increased from 0.1 × 10^6^ to 0.53 × 10^6^ and RBL MCs cells alone have increased from 0.1 × 10^6^ to 0.39 × 10^6^ in 48 h (Figure 1B) whereas EL4 cells co-cultured with RBL MCs, calculating cell numbers at 48 h from the IgE staining percentages shown in the Figure 1A, EL4 cell numbers were found to be 0.84 × 10^6^ and RBL MC numbers were 0.35 × 10^6^ (Figure 1B). L1210 showed almost equal proportions of stained and unstained cells or no change in respective ratios of MCs and L1210 (50.78–47.87%). From the above observations RBL MCs had growth inhibitory effect on YAC-1 cells, growth promoting effect on EL4 cells, and no effect on growth of L1210 cells.

The results suggested that RBL MCs may affect tumor cells when in direct contact. To further assess the cytotoxic/proliferative role MCs on tumor cells, MTT assay was performed to investigate the metabolic activity of the tumor cells (Figure 1C). MTT assay showed significant decrease in absorbance of YAC-1 cells co-cultured with RBL MCs at 12, 24, and 48 h, respectively, as compared to the YAC-1 alone (Figure 1C). On the other hand, RBL MCs co-cultured with EL4 showed opposite result in comparison to YAC-1. EL4 showed significant increase in metabolic activity at 12, 24, and 48 h when RBL MCs and EL4 cells were co-cultured as compared to the EL4 alone. L1210 cells showed no change in absorbance values. These results suggested that RBL MCs inhibited the metabolic activity of YAC-1 cells, enhanced the metabolic activity of EL4 cells and had no effect on metabolic activity of L1210 cells.

We further co-cultured the RBL MCs with tumor cells in a transwell plate and observed the cell recovery after 48 h. The cell viability of only YAC-1 cells showed a significant decrease upon culture with MCs (Figure 1D). Comparing the direct and indirect effect on the YAC-1 cells in percentages, it is 45% reduction cell growth in direct contact whereas only 20% reduction in case of indirect contact (transwell assay). These results suggested that MCs can affect tumor cells mainly through direct contact than indirect contact.

### Mast Cell Secretion in Presence of YAC-1 Tumor Cells

**Figure 2:**
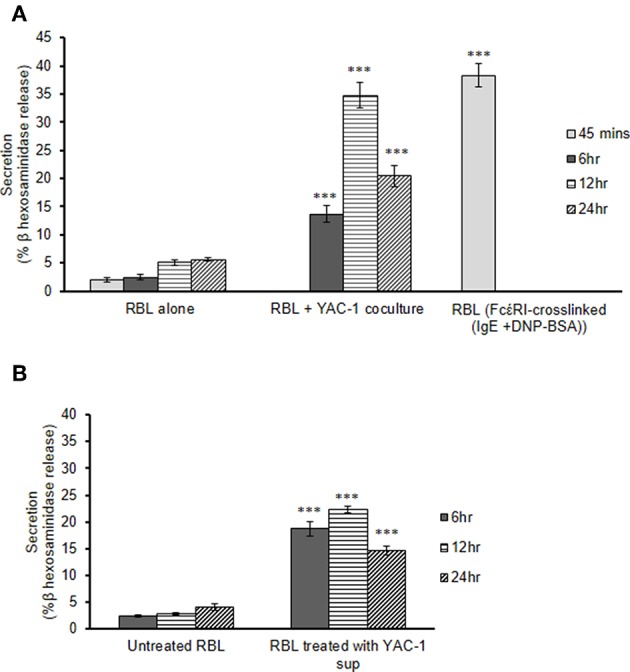
MC secretion in presence of YAC-1 tumor cells. **(A)** Secretion of MC in co-culture with YAC-1 cells for 6, 12, 24 h. Secretion is plotted as % β hexosaminidase release. RBL MC secretion by FcέRI crosslinking for 45 min has been plotted as a positive control. **(B)** Secretion of MC when treated with supernatant of YAC-1 cells for 6, 12, 24 h. Secretion is plotted as % β hexosaminidase release. Data are expressed in comparison to resting cells and are the means ± SEM of three separate experiments. Data was analyzed using *t*-test ^***^P < 0.0005.

To study the role of tumor cells or tumor microenvironment on the mast cells, we further co-cultured the RBL MCs with YAC-1 tumor cells in a 48 well plate and collected the supernatant after 6, 12, and 24 h of incubation. From the supernatant we performed β-hexosaminidase release assay and observed that mast cells are activated and are degranulating in the presence of YAC-1 tumor cells. 13.6, 34.8, 20.47% of β-hexosaminidase was released from RBL mast cells when co-cultured with YAC-1 tumor cells for 6, 12, and 24 h (Figure 2A). We have also studied the impact of tumor cell conditioned media on the degranulation of RBL mast cells. For that, we have cultured YAC-1 tumor cells in a 48 well plate for 6, 12, and 24 h and the supernatants were then used to stimulate mast cells for 6, 12, and 24 h. 18.7, 22.26, 14.65% of β-hexosaminidase was released from RBL mast cells when stimulated with YAC-1 tumor cells supernatant for 6, 12, and 24 h (Figure 2B), respectively. These results suggested that tumor cells in direct contact with MCs stimulate MCs around 13% more than the indirect contact. We found that the secretion in response to tumor cell contact is similar to IgE cross linking as shown in Figure 2A. Hence for further experiments we took RBL MC Supernatant after FcέRI crosslinking to obtain MC mediators.

### Early Phase MC Mediators Inhibit Growth of YAC-1 Tumor Cells

**Figure 3:**
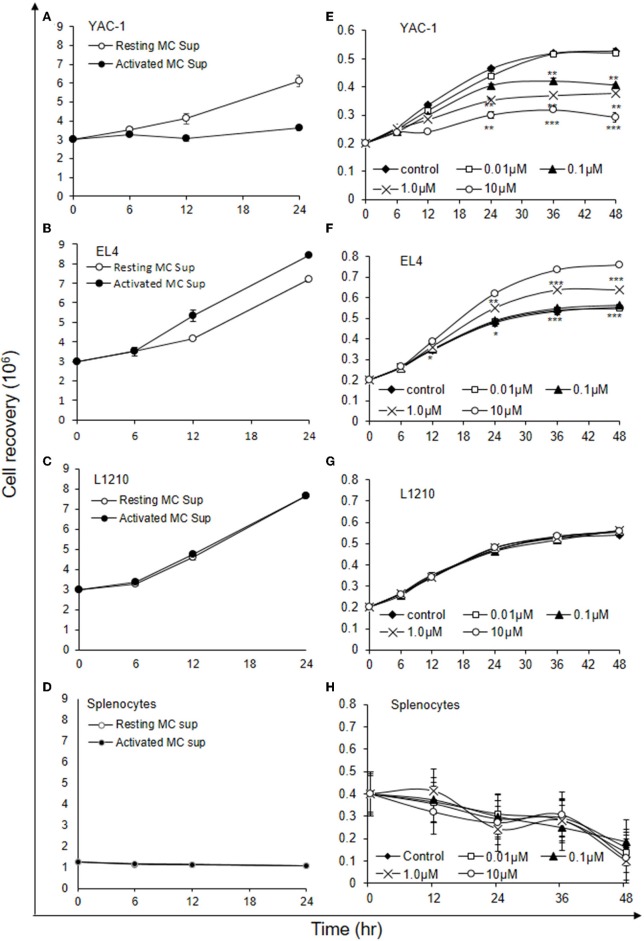
Indirect effects of MC mediators on the growth of tumor cells (cell recovery). **(A–D)** For the studies assessing the effect of MC mediators on cell growth, tumor cells or splenocytes (from C57BL/6 mouse) were cultured in 24 well plate and after 12, 24, and 48 h of the treatments, viable cells were counted using trypan blue staining and hemocytometer. **(E–H)** For the studies assessing the effect of Histamine on cell growth, tumor cells or splenocytes (from SWISS mouse) were cultured in 24 well plate and after 6, 12, 24, 36, and 48 h of the treatments, viable cells were counted using trypan blue staining and hemocytometer. Data are expressed in comparison to control cells (cells treated with resting MC sup) and are mean ± SEM of three separate experiments, each of which was performed in triplicate, Data was analyzed using *t*-test, ^*^p < 0.05, ^**^p < 0.005, ^***^p < 0.0005.

**Figure 4:**
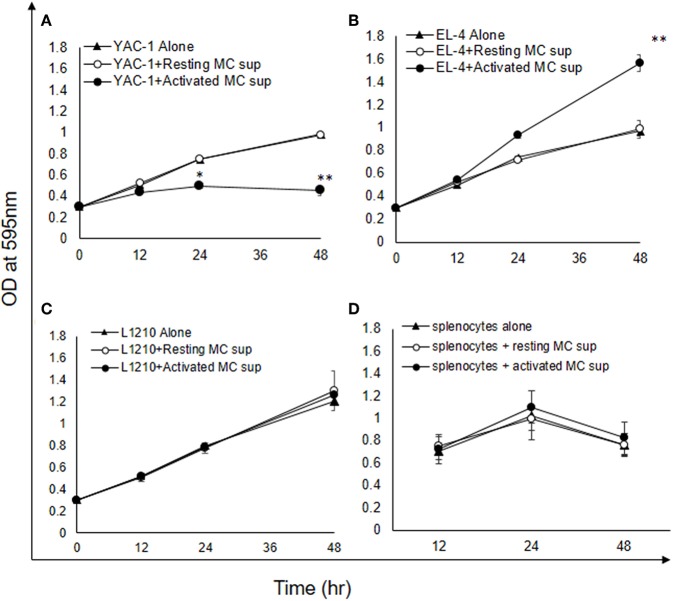
Indirect effects of MC mediators on the growth of tumor cells (MTT assay). **(A–D)** Indirect effect of MCs on tumor growth was assayed using MTT assay. 0.02 × 10^6^ tumor cells or 1.5 × 10^6^ splenocytes (from C57BL/6 mouse) were treated with MC mediators for 6, 12, and 24 h and after incubation MTT was added and blue formazan crystals trapped in cell were dissolved using DMSO and absorbance was taken at 595 nm using plate reader. Data are expressed in comparison to control cells (cells treated with resting MC sup) and are mean ± SEM of three separate experiments, each of which was performed in triplicate, Data was analyzed using *t*-test, ^*^p < 0.05, ^**^p < 0.005.

As shown above MCs could have direct or indirect effect on tumor cells. MCs might affect tumor cells function with their released mediators. To investigate the role of MCs mediators, tumor cells were treated with resting MC's supernatant obtained without IgE crosslinking as well as activated MC's supernatant obtained from crosslinking of IgE bound to FcεRI receptors by allergen (DNP-BSA-100 ng/ml). YAC-1 cells when treated with MC mediators showed a significant inhibition in cell viability, as assessed by hemocytometer cell counting after trypan blue staining (Figure 3A). Treatment of EL4 cells with activated MCs mediators resulted in a significant increase in EL4 cell growth (Figure 3B). When these tumor cell lines were treated with resting MCs' mediators (no crosslinking/no sensitization), no significant changes in cell growth were observed in either of the cell lines. Whereas, in L1210 cell line and splenocytes isolated from mouse, no alterations in cell growth were detected on incubation with activated and resting MC supernatants (Figures 3C,D). Similar observation was also obtained in MTT assay experiment (Figures 4A–D).

Histamine being a major MC early phase mediator (21), its effect at various concentrations was then investigated on tumor cells. YAC-1 cells when treated with histamine showed a significant inhibition in cell viability with increasing concentration, as assessed by hemocytometer cell counting after trypan blue staining (Figure 3E). Treatment of EL4 cells with increasing concentration of histamine showed significant increase in EL4 cell growth (Figure 3F). The effects were prominent at higher concentration of histamine i.e., at 1 and 10 μM. In L1210 cell line and splenocytes isolated from mouse, no alterations in cell growth were observed on incubation with histamine (Figures 3G,H). The results suggest that MC mediators generated *in vitro* and histamine are exerting similar effects on tumor cells.

### MC Mediators' Treatment Induced Apoptosis in YAC-1 Tumor Cells

**Figure 5:**
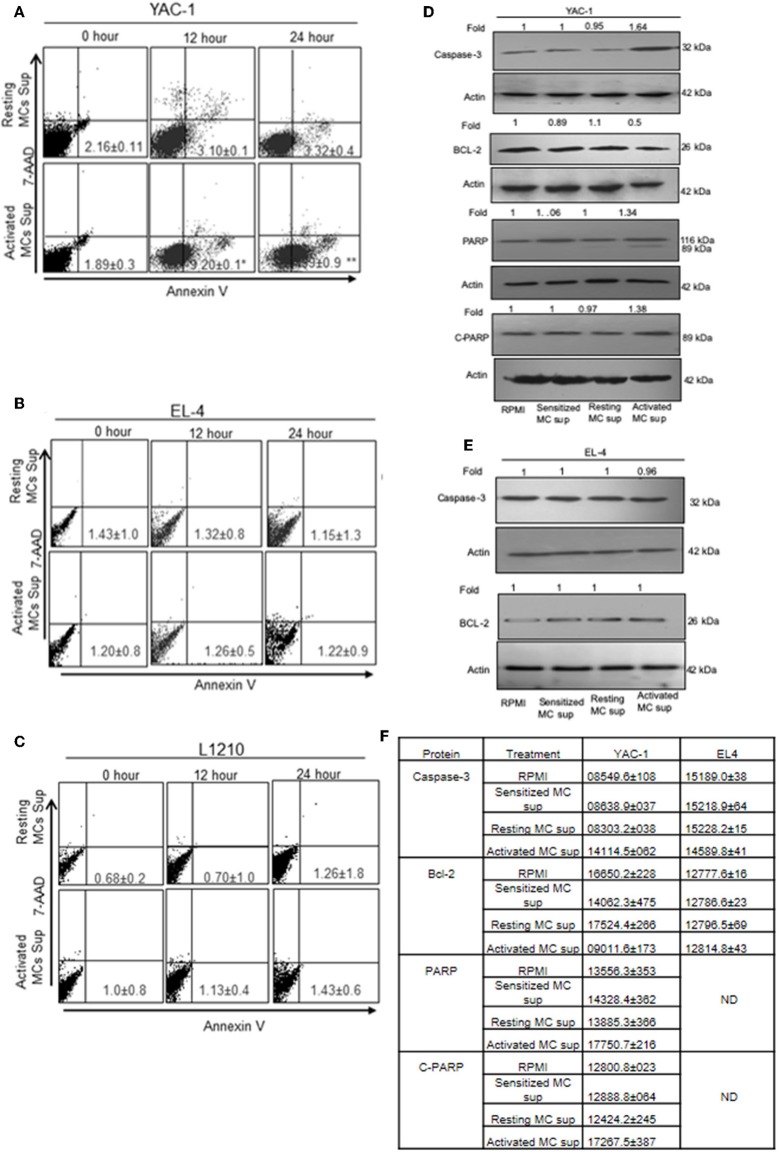
The apoptotic effect of MC mediators on tumor cells. Tumor cells were treated with MC mediators for 0, 12, and 24 h. At the end of treatments, total cells were collected and stained with annexin V/7AAD and analyzed for apoptotic cell population as mentioned in section Materials and Methods. Representative histogram from three sets of experiments has been represented for YAC-1 cells **(A)**, EL4 **(B)**, and L1210 **(C)**. Numbers represent the apoptotic cells. In similar treatments as detailed above, total cell lysates were prepared at 24 h. SDS-PAGE and western blot analysis were performed for Caspase-3, Bcl-2, total PARP, and as well as cleaved PARP for YAC-1 **(D)**, and EL4 **(E)**. Membranes were stripped and re-probed with anti-beta-actin antibody to ensure equal protein loading. Numbers on top of the bands represent fold changes in band intensity as compared to control as determined by densitometric analysis of the bands and corrected for beta-actin loading control for western blot. **(F)** Means ± SEM band intensities are shown in the table. Data are expressed in comparison to control cells (untreated cells i.e., cells with RPMI) and are the means ± SEM of three separate experiments, each of which was performed in triplicate, Data was analyzed using *t*-test. ^*^p < 0.05, ^**^p < 0.005.

To further elucidate the mechanism of growth inhibition or promotion, we examined the proportion of apoptotic and necrotic cells as described in Materials and Methods. The quantitative apoptotic/necrosis cell death assay was done using Annexin/7AAD staining through flow cytometry analysis. YAC-1 cells showed increased proportions of apoptotic cells when monitored on increasing time points upon treatment with MC mediators. The proportions of early apoptotic cells were found to be 1.89 ± 0.3, 9.20 ± 0.1, and 27.99 ± 0.9% at 0, 12, and 48 h, respectively (Figure 5A). Whereas, no necrotic cells were observed upon the treatment. Therefore, apoptosis related proteins like Capsase-3, Bcl-2, total PARP, and cleaved PARP were evaluated after 24 h of treatment. The expression of Caspase-3 was increased by 1.64-folds (64% increase), Bcl-2 was decreased by 0.5-folds (50% decrease) and total and cleaved PARP were increased by 1.34-folds (34% increase) and 1.38-folds (38% increase) (Figure 5D), respectively.

In EL4 and L1210 cells, no signs of apoptotic response were observed in flow cytometric analysis. No significant change was observed in apoptotic and necrotic cells in EL-4 and L1210 on treatment with resting and activated MC supernatant at any time point (Figures 5B,C). The expression of Caspase-3 and, Bcl-2 in EL4 cells showed no change (Figure 5E). The mean ± SEM band intensities of three independent experiments are shown in Figure 5F for YAC-1 and EL4 cells.

### MC Mediator Treatment Induced Cell Cycle Arrest in YAC-1 Cells

**Figure 6:**
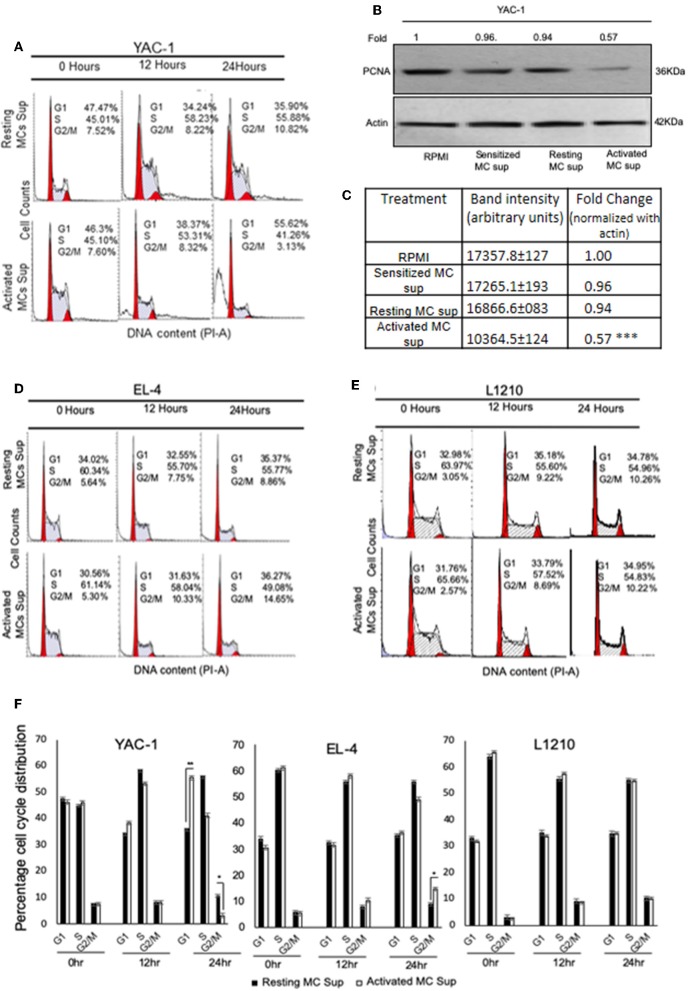
Effect of MC mediators on cell cycle of tumor cells. Tumor cells were treated with resting and activated MC supernatants for 0, 12, and 24 h. At the end of treatments, cells were collected and analyzed using flow cytometry and MODFIT software for cell cycle phase distribution as detailed in Materials and Methods. Representative histogram [The red peaks shown are of G0/G1 (left) and G2/M (right) and the area between the two peaks are the cells in S phase.] from three set of experiments has been represented for **(A)** YAC-1 cells, **(D)** EL4, and **(E)** L1210. SDS-PAGE and western blot analysis were performed for PCNA for YAC-1 **(B)**. Numbers on top of the bands represent fold changes in band intensity as compared to control as determined by densitometric analysis of the bands and corrected for beta-actin loading control for western blot. **(C)** Means ± SEM band intensities of PCNA are shown in the table. **(F)** Represents the percentage of tumor cells obtained at various phases of cell cycle on treatment with MC mediators for 12 and 24 h. Data are expressed in comparison to cells treated resting MC sup for cell cycle experiment and untreated cells for PCNA and are the mean ± SEM of three separate experiments, each of which was performed in triplicate, Data was analyzed using *t*-test ^*^p < 0.05, ^**^p < 0.005, ^***^p < 0.0005.

Another reason for growth inhibition or promotion could be alterations in cell cycle/cell division. Hence, we performed cell cycle analysis for these tumor cells after treatment with MC mediators. Flow cytometric study of cell cycle distribution proposed that retarded cell growth of YAC-1 cells was due to accumulation at G0-G1 phase of cell cycle (Figure 6A). This was also supported by western blot analysis of PCNA. The dividing animal cells have PCNA (proliferating cell nuclear antigen) in their nucleus which suggests it to have a function in cell cycle regulation or DNA replication (37). PCNA expression was found to be decreased to 0.57-folds (43% decrease) in YAC-1 cells treated with MC mediators for 24 h as shown in the Figure 6B and band intensities are shown in Figure 6C. While in EL4 cells, there was increase in cells at G2/M phase of cell cycle i.e., 7.75 ± 0.5 and 8.86 ± 1.0% with resting MC supernatant and 10.33 ± 0.2 and 14.65 ± 1.0% with activated MC supernatant (Figure 6D) indicating a slight increase in cell division. In case of L1210 there was no change in cells at any phase of cell cycle (Figure 6E) with MC mediator exposure. Distribution of cells in various stages of cell cycle is shown in Figure 6F.

### Growth Inhibition of YAC-1 Is Due to Effect on Survival Related Genes

**Figure 7:**
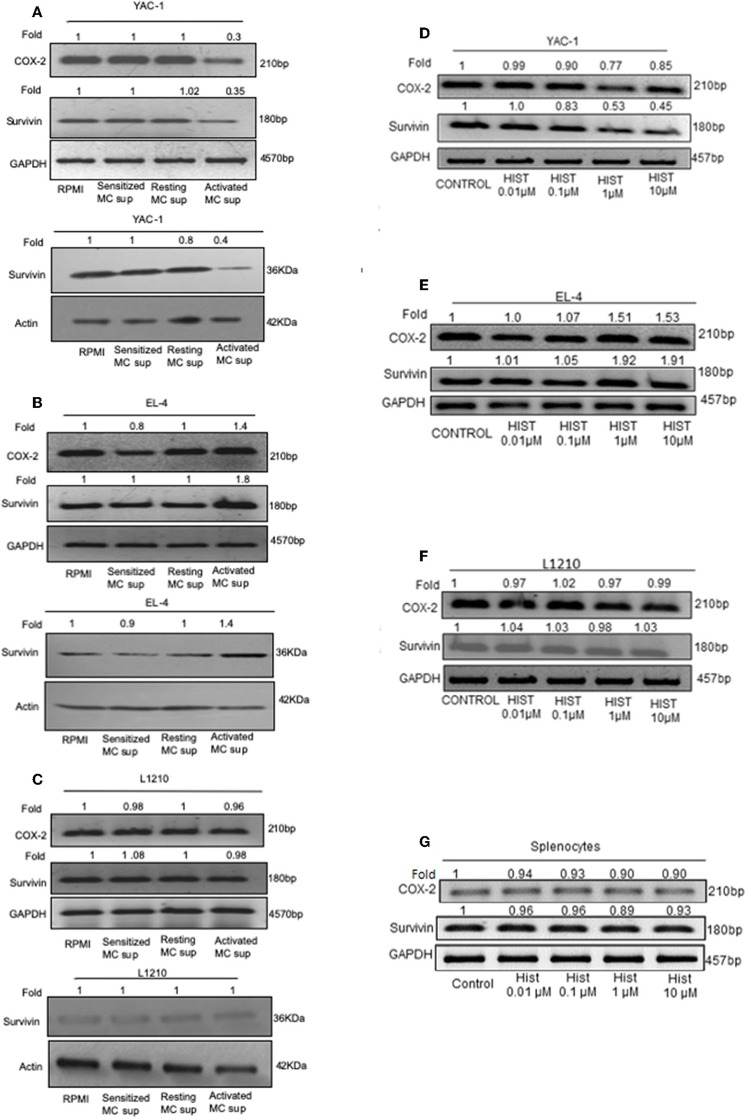
Effect of MC mediators on expression of genes important for survival of tumor cells. 1 × 10^6^ tumor cells were cultured in 60-mm dishes and treated with MC mediators or different concentration of histamine (0.01, 0.1, 1, 10 μM). After 24 h of treatment, RNA and protein were isolated as described in section Materials and Methods. Semi-quantitative RT-PCR for *COX-2* and *Survivin* was performed for YAC-1 **(A,D)**, EL4 **(B,E)**, L1210 **(C,F)**, and splenocytes **(G)** along with the loading control, *GAPDH*. Western blot analysis was done for survivin after MC mediators treatment, YAC-1 **(A)**, EL4 **(B)**, and L1210 **(C)** where in each well 40 μg of protein were loaded. The intensity of the resulting bands was evaluated by densitometry analysis and the Survivin/actin ratio was reported with loading control beta-actin. Representative gels and immunoblots of results obtained from three independent experiments are shown. The numbers on the top of the bands represent fold changes in band intensity as compared to control as determined by densitometric analysis of bands and corrected for *GAPDH* and beta-actin. The experiment was repeated thrice.

These results so far suggested that MC mediators played a dual role in affecting the tumor cell growth, inhibiting the proliferation of YAC-1 cells and increasing the proliferation of EL4 cells. To further study the mechanisms involved, the mRNA expression of endogenous gene *COX-2, Survivin* and also protein expression of Survivin were investigated. Both these genes are essential for cell growth (38, 39). Results showed a significant decrease in expression of *Survivin* at mRNA as well as protein level in YAC-1 cells. It was found to be reduced to 0.35-folds (65% decrease) at mRNA level and reduced to 0.4-folds (60% decrease) at protein level. Reduction to 0.3-fold (70% decrease) in mRNA expression of *COX-2* was observed in YAC-1 cells upon treatment with MC activated supernatants (Figure 7A). While in EL4 cells, *Survivin* expression was increased to 1.8-folds (80% increase) at mRNA level and increased to 1.4-folds (40% increase) at protein level. Increment of 1.4-folds (40% increase) in mRNA expression of *COX-2* was observed in EL4 cells (Figure 7B). This result suggested that the decrease in cellular viability of YAC-1 cells was due to differential regulation of *Survivin* and *COX-2*. No such differential expression was observed in L1210, both at mRNA as well as protein level (Figure 7C). There was no effect of MC mediator exposure on expression of *Survivin* or *COX-2* in L1210.

As histamine is the prime mediator of mast cells, we explored the effect of histamine on Survivin and COX-2 expression in tumor cells upon histamine treatment. Results were found to be similar as MC mediator's treatment. Results showed a significant reduction to 0.45-folds (55% decrease) in expression of *Survivin* at mRNA level in YAC-1 cells and reduction to 0.85-fold (15% decrease) in mRNA expression of *COX-2* was observed in YAC-1 cells upon treatment with increasing concentration of histamine (Figure 7D). While in EL4 cells, *Survivin* and *COX-2* expression was increased to 1.91-folds (91% increase) and 1.53-folds (53% increase), respectively, at mRNA level (Figure 7E) on treatment with histamine. In both, YAC-1 and EL4, the effects were more prominent at higher concentration of histamine i.e., at 1 and 10 μM. No such differential expression was observed in L1210 and mouse splenocytes at mRNA level of *Survivin and COX-2* (Figures 7F,G). The band intensities are shown in Supplementary Tables 3–5. Therefore, the results suggests that histamine also exerts a differential effect on proliferation related molecules of YAC-1 and EL4 cells, no effect on L1210 cells and on mouse splenocytes.

### Exposure to MC-Mediators Affects Mitochondrial Health of YAC-1 Tumor Cells

**Figure 8:**
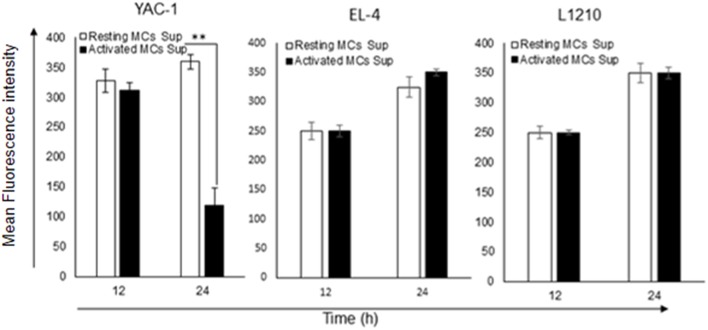
Effect of MC mediators on mitochondrial health of tumor cells. To check the mitochondrial potential of tumor cells, 0.2 × 10^6^ each tumor cells were treated with MCs mediators. After 12 and 24 h of treatment, cells were harvested as described in methods and materials. Total cells were collected and stained to assess mitochondrial membrane potential with JC-1 and analyzed by flow cytometer for YAC-1, EL4, and L1210. Bar-diagrammatic representation of respective time point, Data are expressed in comparison to cells treated with resting MC sup and are the means ± SEM of three separate experiments, each of which was performed in triplicate, Data was analyzed using *t*-test ^**^p < 0.005.

To investigate whether the growth inhibitory effect was also linked with an alteration in the mitochondrial health, mitochondrial membrane potential was quantified by JC-1 uptake and analyzed by flow cytometry. MC mediators caused a reduction in mitochondrial potential in YAC-1 cells (Figure 8). The result indicates that MC mediators can cause the lysis or fusion of mitochondria along with the decline of total surface area. In comparison, EL4 and L1210 cells showed no significant change in mitochondrial health.

### Expression of Histamine Receptors and Antagonist Treatment on Tumor Cells

**Figure 9:**
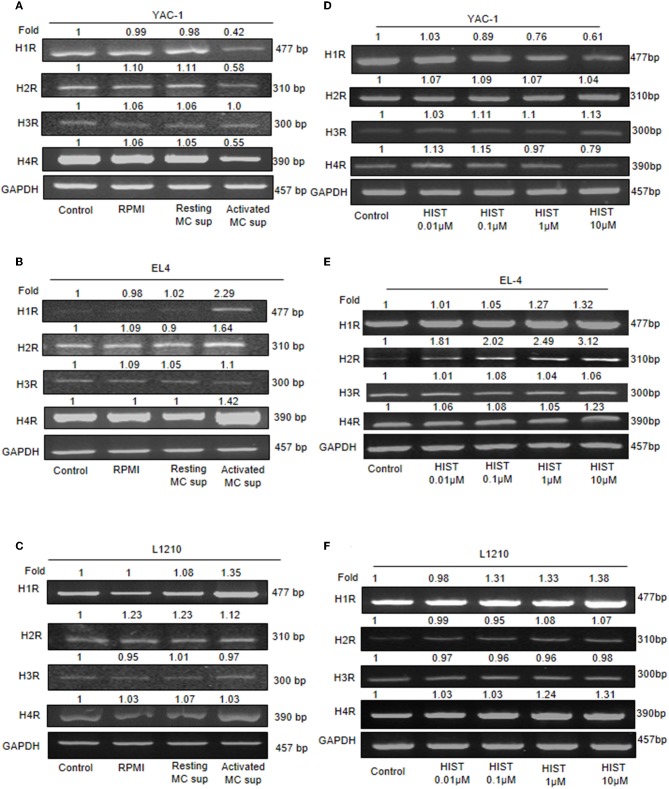
Expression of histamine receptors on Tumor cells. 1 × 10^6^ tumor cells were cultured in 60-mm dishes and treated with MC mediators or histamine (10 μM) for 24 h. Cells were harvested and then washed with PBS and RNA was isolated using Trizol- chloroform method and cDNA was synthesized. Gene specific primers for H1, H2, H3, and H4 receptors were used to amplify cDNA. mRNA expression was detected for YAC-1 **(A,D)**, EL4 **(B,E)**, and L1210 **(C,F)** by RT-PCR analysis. Representative gels results were obtained from three independent experiments are shown. The numbers on the top of the bands represent fold changes in band intensity as compared to untreated cells (control) as determined by densitometric analysis of bands and corrected for GAPDH. Representative gels from experiments repeated thrice.

Histamine being a major MC mediator and previous studies have shown its effects on tumor growth (22, 40, 41). To validate the involvement of histamine receptors in the heterogeneous effect of MC mediators on tumor cell lines, we analyzed the endogenous expression of all four histamine receptor (H1R–H4R). RT-PCR analyses showed that all tumor cells expressed H1, H2, H3, and H4 receptor mRNA, suggesting that histamine may effectively trigger response in tumor cells through any of its receptors. Treatment of YAC-1 with MC mediators resulted in significant down regulation of mRNA for H1R, H2R, H4R, and no effect in H3R as shown in the Figure 9A. For EL4, up-regulation in H1R, H2R, and H4R were observed, while no effect was seen in H3R (Figure 9B). For L1210 cells, no change in receptor expression pattern was observed on treatment with MC mediators (Figure 9C). To validate the response is because of histamine, we used 0.01, 0.1, 1, 10 μM of histamine to treat the tumor cells and looked at its effect on the histamine receptor expression profile. Treatment of YAC-1 with histamine resulted in significant down regulation of mRNA for H1R, H4R, upregulation of H3R and no effect in H2R as shown in the Figure 9D. For EL4, up-regulation in H1R, H2R, and H4R were observed, while no change was seen in H3R (Figure 9E). Whereas, for L1210 cells, H1R and H4R was upregulated and no change in other receptors expression pattern was observed on treatment with histamine (Figure 9F). The band intensities are shown in Supplementary Tables 6, 7.

**Figure 10:**
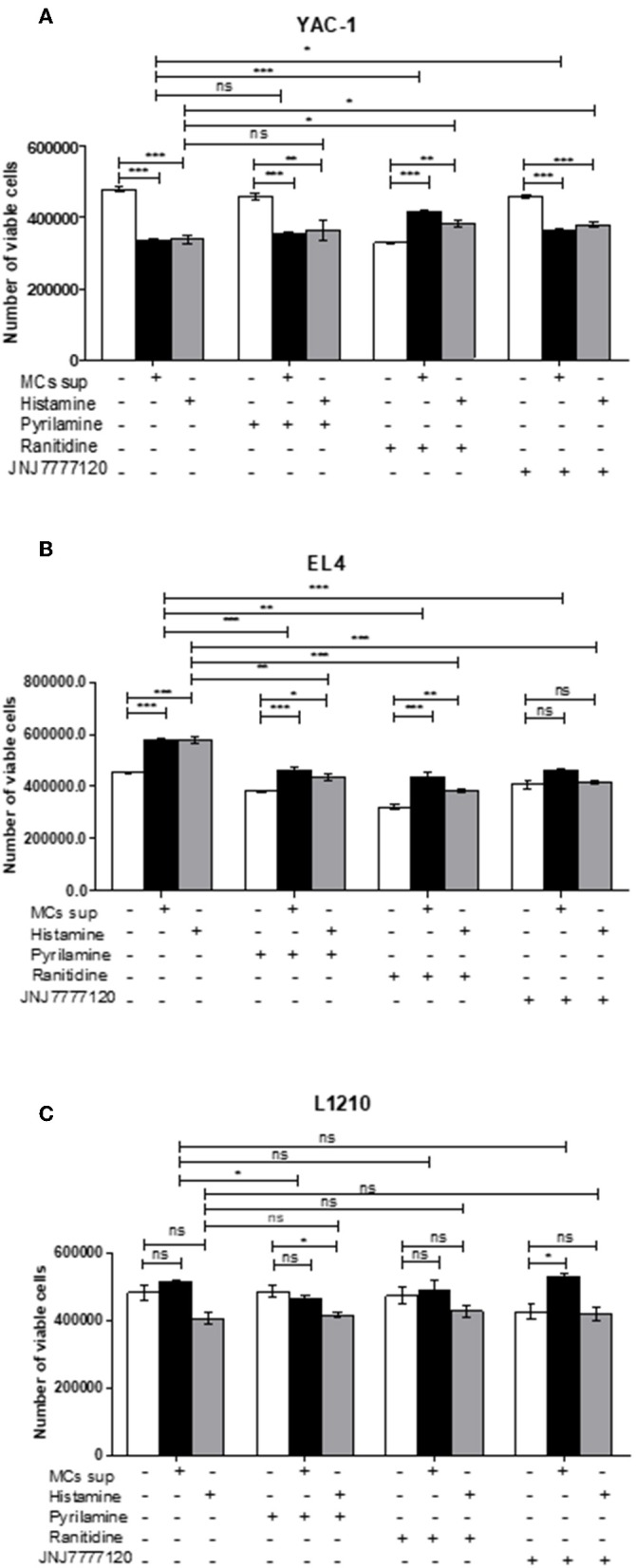
Effects of histamine receptor antagonists on viable cell recovery of tumor cells. Tumor cell lines were cultured with MC mediators or histamine (10 μM) for 24 h in presence and absence of histamine antagonists. The effects of pyrilamine (10 μM), ranitidine (10 μM) and JNJ7777120 (10 μM) alone or in combination with MC mediators for 24 h YAC-1 **(A)**, EL4 **(B)** and L1210 are shown **(C)**. Cell growth was measured by using trypan blue staining and hemocytometer. Data are expressed as the percentage of control cells (untreated cells) and are the means ± SEM of three separate experiments, each of which was performed in triplicate. Data was analyzed using *t*-test, ^*^p < 0.05, ^**^p < 0.005, ^***^p < 0.0005, ns, not significant.

Further, the selective histamine receptor antagonists, pyrilamine (H1R), ranitidine (H2R), and JNJ7777120 (H4R), respectively, were used in combination with MC mediators or histamine and cell growth patterns assessed by trypan blue staining and hemocyotmeter. Pyrilamine given in combination with MC mediators led to no change in cell recovery of YAC-1 cells (Figure 10A), a significant decrease in cell recovery of EL4 cells (Figure 10B), and a significant decrease in recovery of L1210 cells (Figure 10C). Ranitidine in combination with MC mediators caused increase in cell recovery of YAC-1 cells (Figure 10A) and decrease in cell recovery of EL4 cells (Figure 10B). Also JNJ7777120 given in combination with MC mediators caused increase in cell recovery of YAC-1 cells (Figure 10A) and decrease in cell recovery of EL4 cells (Figure 10B). Therefore, the results suggest that MC mediators triggered the inhibition of cell growth in YAC-1, possibly through the H2 and H4 receptor, while they increased cell growth in EL4 cells, possibly through the H1, H2, and H4 receptors. L1210 cells on the other hand seem more or less inert to MC mediators. To validate the effect is because of histamine present in MC mediators, we used histamine 10 μM (Sigma Aldrich) with the selective histamine receptor antagonists as mentioned above to treat the tumor cells and cell growth patterns were assessed by trypan blue staining and hemocyotmeter. Pyrilamine given in combination with histamine led to no change in cell recovery of YAC-1 cells (Figure 10A), a significant decrease in cell recovery of EL4 cells (Figure 10B), and no change in recovery of L1210 cells (Figure 10C). Ranitidine in combination with histamine caused increase in cell recovery of YAC-1 cells (Figure 10A), decrease in cell recovery of EL4 cells (Figure 10B), and no change in recovery of L1210 cells (Figure 10C). Also JNJ7777120 given in combination with histamine caused increase in cell recovery of YAC-1 cells (Figure 10A), decrease in cell recovery of EL4 cells (Figure 10B), and no change in recovery of L1210 cells (Figure 10C). The effects shown by MC mediators are similar to the effects shown by histamine, therefore we can say that histamine is the major MC mediator showing effects on tumor cell proliferation through histamine receptors H1–H4.

## Discussion

The progression of tumor is a result of activation or inactivation of different genes (42), which have been regarded as master regulators. The genetic and epigenetic alterations have determined the cancer microenvironment, as a prime cause of its development (43). Tissue microenvironment which consist of blood and lymphatic vessels, some important immune cells, extracellular matrix (ECM), and fibroblasts, plays a chief and crucial role in maintenance of homeostasis and also provides a barrier to tumorigenesis (44). In recent times, clinical and experimental studies have shown the role of inflammatory cells like MCs in cancer. All the functions of MC depend on the release of proinflammatory mediators upon activation. There is a report stating that mediators reside in distinct secretory granule subsets whose exocytosis is regulated by different fusion mediator proteins (30). The main mechanism of MC activation can be either IgE dependent or independent (45–47), which overall depends on the activation of surface receptors expressed by MCs. MCs are also a component of cancer microenvironment, the role of which is complex and poorly understood. Several studies have shown that MCs either promote or suppress tumor growth and development whereas in few cases they are inert. The defined role of MC in tumor is still unclear and it depends on both localization of cell, stage of tumor, and type of tumor (48).

Since, MCs have already been identified in microenvironment of different cancers; therefore, studying role of MCs in different tumors will improve our understanding and further identification of signaling pathways leading to heterogeneous role of MCs in tumors. YAC-1, EL4, and L1210 are rodent tumor cells representing Lymphomas and Leukemia that were used in the current study. Direct as well as indirect (MC mediators generated *in vitro* or histamine) contact of MCs with tumor cells showed reduction/increase in cell viability starting as early as from 12 h in a time dependent manner. The co-culture experiment of tumor cells with MCs has shown 45% decrease in YAC-1 cell population whereas 55% increase in EL4 cell population and interestingly no change in the RBL MC population. These results suggest that MCs can influence tumor cell activity through direct cell to cell interactions. Apart from direct contact, we have studied the indirect contact of MC with tumor cells with transwell assay. This assay was important to know if tumor cells or MC have any interaction through their supernatants. The result suggested that there is 20% decrease in YAC-1 in transwell assay but no significant change in EL4 and L1210. There was some effect on YAC-1 cells but lesser than in direct contact with MCs. Further to check whether tumor cells influence MCs to secrete in tumor microenvironment and to validate the indirect effects between the two cells, we co-cultured YAC-1 along with MCs and collected the supernatant and lysates at 6, 12, and 24 h and checked for β-hexosaminidase release. Maximum MC secretion in co-culture was seen at 12 h which is around 37% which is comparable to the positive control i.e., RBL secretion by IgE receptor crosslinking (activated MCs). We also checked the secretion of RBL MCs by only treating with YAC-1 supernatants for 6, 12, and 24 h. There was around 23% release at 12 h. It is known that in resting state also MCs do secrete some mediators (49) though much less than what is released on activation. Also maybe some soluble factors (32) from YAC-1 may enhance this basal secretion as shown in Figure 2B. Therefore, we then used MC mediators generated by crosslinking FcέRI for 45 min. In correlation with direct role of MCs, MC mediators also showed the similar effect on tumor cells, decreased the proliferation of YAC-1 cells, increased proliferation of EL4 and no effect on L1210. Further we studied the effect of histamine as it is the prime mediator released by MCs (21). The physiological histamine concentration in solid tumors like breast cancers has been reported to be more than the normal tissues (50). Increase in histamine concentration is the consequence of MCs infiltration in the tumor tissues (50). There are studies reporting that histamine modulates the invasive phenotype of tumor cells in a dose dependent manner in solid cancers like pancreatic and breast cancers (51, 52). In order to study the involvement of histamine in increase or reduction of tumors, we treated YAC-1, EL4, L1210, and normal mouse splenocytes with histamine at four different concentrations ranging from 0.01 to 10 μM. The effects of histamine were parallel to those of MC mediators as histamine treatment also caused a decrease in cell proliferation of YAC-1 cells, increased the proliferation of EL4 cells, and had no effect on L1210 and normal mouse splenocytes. Further studying the mitochondrial membrane potential showed there's overall decrease in membrane potential of active mitochondria inYAC-1 cells which could indicate mitochondrial fusion in these cells.

The key event involved in tumor development is increased cell proliferation. Survivin is an anti-apoptotic protein generally upregulated in cancer cells, and also known to provide capability to survive with the inhibition of the activation of apoptosis cascade (53). qRT-PCR analysis showed significantly decreased expression of Survivin and COX-2 in YAC-1 cells after treatment with MC mediators or histamine. MC mediators or histamine treatment on EL4 cells enhanced Survivin and COX-2 expression but no effect was seen in L1210 cells. Our study shows that MC mediator's leads to decrease in Survivin at both mRNA as well protein levels supporting the notion that reduction in YAC-1 cell survival on treatment with MC mediators could be umpired via apoptosis. Initiation of apoptosis is generally linked with arrest in cell cycle providing the cells with some time to adjust with the changes in exterior environment as well as changes happening intracellularly (54). Thus, our finding of YAC-1 population to increase in sub-G0/G1 indicates, cells going for cell death that may involve apoptosis. We then explored the proportion of cells undergoing apoptosis and changes associated with at molecular level following treatment with MC mediators. As anticipated, increase in number of apoptotic cells was time dependent as analyzed through flow cytometry. Increase in cells dying by apoptosis was accompanied by a decrease in total PARP and increase in cleavage of Caspase-3. The MC mediator's induced Caspase-3 activation can be confirmed by the generation of the c-PARP (cleaved PARP) which is the target of Caspase-3 (55). Caspase activation symbolizes apoptosis process; therefore the result of this study suggests that reduction in YAC-1 cells survival following MC mediator's treatment is probably linked to the activation of Caspase-3 to induce the apoptosis cascade. Collectively, G0-G1 phase arrest in cell cycle and Caspase-3 mediated apoptosis after treatment with MC mediators indicated the role of MCs in tumor cell death.

A large number of receptors involved in cell proliferation have been investigated including histamine receptors (56). The differential outcome could be due to histamine receptor expression on tumor cells upon treatment of MC mediators, the cellular responses by Histamine are mainly mediated through its four receptors, H1-H4 histamine receptors, and their expression can be changed or altered in various diseases, and also varies between different tissue types (57). The histamine receptors that are generally found in tumor cells are linked with numerous signaling pathways (58–60). Also, the presence of histamine receptors in various tissues including tumors propose new roles for histamine which lead to new outlooks in histamine pharmacology research (61–63). Recent findings have indicated that histamine-mediated biological processes in tissues and cell lines are mediated by histamine receptors. Histamine receptors have been reported to control various biological processes like cell proliferation, apoptosis, metastatic potential in malignant cells etc. (64, 65). Histamine exerts both proliferative and proangiogenic effect via activation of its corresponding receptors in cancer cells (56). Thus, we further investigated the detailed role of histamine receptors in cell proliferation. Decrease in expression of histamine receptors H1R, H2R, and H4R was observed upon treatment of MC mediators in YAC-1 cells. While EL4 cell line showed the enhanced expression of H1R, H2R, H4R while no change was seen in H3R in all the three cell lines. This complete observation indicated that histamine receptor expression plays a vital function in cell proliferation. To validate the above finding, we then treated the tumor cells with Histamine and looked at the expression profile of histamine receptors. We found that with histamine, H1R and H4R are decreased upon treatment in YAC-1 cells. While in EL4, upon histamine treatment the receptors H1R, H2R and H4R are upregulated. Proliferation profile upon treatment with different histamine receptor antagonist (Pyrilamine-H1R antagonist, Ranitidine-H2R antagonist, and JNJ7777120-H4R antagonist) was investigated in YAC-1 cells, the growth inhibitory function of MCs mediators or histamine was reversed upon addition of Ranitidine or JNJ7777120 with MC mediators or histamine. While in case of EL4, MC mediators or histamine showed enhanced proliferation. Treatment of cells with the receptor antagonist pyrilamine, ranitidine, and JNJ77120 in combination with MC mediators showed growth inhibition. Therefore, it is concluded that MC mediators initiated the reduction in cell growth in YAC-1, probably through the H2 and H4 receptor, while it enhanced cell growth in EL4 cells, perhaps through the H1, H2, and H4 receptors. The effects on histamine receptors exerted by MC mediators could be due to the histamine present in it as the results obtained with histamine are parallel to the results exerted by MC mediators. Our study correlates the effects obtained using MC mediators and effects using Histamine but there could be some other mediators as well, present in the MC secretion that may also be important for the effects seen in this study. Here we are emphasizing on the histamine receptor expression and its importance in increased/decreased cell proliferation of tumor cells. There may be some other receptors also that are functioning in increase/reduction of cell proliferation. As mast cells release a vast consortium of mediators, the fact cannot be denied that there are some mediators other than histamine that are responsible for the action. Therefore, further studies are required to dig out the mechanism by which MCs are acting upon tumor cells as these cells have always been the part of tumor microenvironment and played a controversial role.

**Figure 11:**
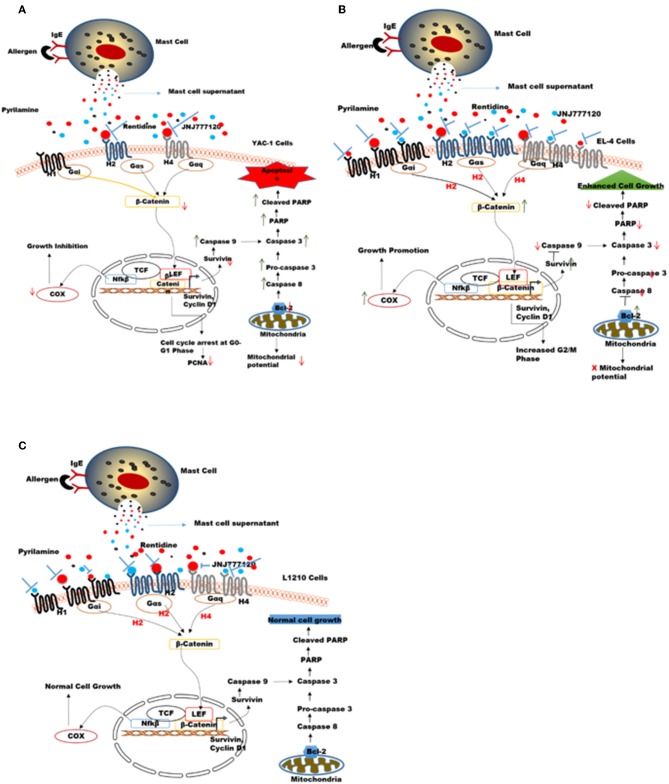
Proposed mechanisms for the role of MC mediators in response to tumor cells. MC mediators may alter hematologic tumor cell survival by modulating the histamine receptor expression differentially in different tumor cell lines, thereby causing opposing effects on downstream signaling events and molecules affecting survival, apoptotic pathway, mitochondrial health, and cell cycle in these tumor cells **(A)** In YAC-1 tumor cells, MCs cause down regulation of histamine receptors H1R, H2R, and H4R receptor. Out of these receptors H2R (inhibited by ranitidine) and H4R (inhibited by JNJ7777120) may be further involved in growth inhibition/reduced survival of YAC-1 cells. MC mediators cause further down regulation of β-catenin pathway. the combined effects lowers the expression level of COX-2, Survivin, Bcl-2, PARP protein, and enhance caspase-3 subsequently leading to cell cycle arrest and apoptosis in tumor cells **(B)** In EL4 cells, MCs cause up-regulation of histamine receptors H1R, H2R, and H4R receptor. Out of these receptors H1R (inhibited by pyrilamine), H2R (inhibited by ranitidine), and H4R (inhibited by JNJ7777120) may be further involved in proliferation and increased survival of EL4 cells. MCs mediators up regulate β-catenin pathway enhancing cell growth. The combined effects increase the expression level of COX-2, Survivin, Bcl-2, and downregulatecaspase-3 subsequently leading to increased cell growth. **(C)** In L1210 cells MCs cause up-regulation of H1R only while H2R and H4R were not affected. Also these receptors may not be involved in growth of these cells. MC mediators do not alter β-catenin pathway subsequently leading no effect on cell growth.

An overall model summarizing the above findings has been proposed in Figures 11A–C. Tumors are variable in different individuals and cancer therapies available nowadays are general not specific to tumor-type. Here, in our study the two cell lines used namely YAC-1 and EL4 are both murine T cell lymphoma cell lines but their origin of cancer is different. We found that MCs inhibited the growth of YAC-1 cancer cell line whereas increased the proliferation of EL4 cancer cell line. It could be due to the difference in expression of histamine receptors on these cell lines as we found that when activated MC mediators are given to these cell lines, the histamine receptor expression on YAC-1 decreased while in EL4 it increased. It could be due to some mediators present in the MC activated supernatant that is responsible for this up regulation or down regulation of these histamine receptors, the cause is still not known. Also the cell line, YAC-1 having less histamine receptors is more susceptible to growth inhibition and cell death when comes in contact with histamine present in activated MC supernatant. Whereas, it is completely opposite in the case of EL4 cell line, which is having more histamine receptors and growth and proliferation increased in its case. Therefore, we can conclude that MCs might be present as sentinel cells in the tumor microenvironment and play a vital part in tumor growth inhibition or progression depending on tumor characteristics and receptors present on it. So, to exploit MCs or their mediators in the tumor therapy, it is important to characterize the tumors and also profile histamine receptors present on the tumor cells. If less histamine receptors are present, MC mediators may help in inhibiting the tumors, in these cases MC mediators or activators can be given to treat the tumors along with the conventional therapy. On the other hand if more histamine receptors are present, some blockers may be given to block the release of mediators from MCs.

The effect of MC mediators on gene transcription through histamine receptor signaling was investigated in this study. It is known that histamine upon binding to its receptors disarms GSK3-β, thus stabilizing β-catenin, and further activating TCF-dependent transcription in many cell types (66). A general feature of histamine receptor interaction is the activation of Wnt/frizzled-like signaling cascade. Not only frizzled-induced Disheveled pathway is involved but also β-catenin pathway is involved in histamine receptor activation (66). Furthermore, the fact that histamine receptor is the G protein coupled receptor, which shows that Gα protein can release β-catenin from cadherins thus activating β-catenin-dependent transcription. Decreased cellular proliferation and increased apoptosis in YAC-1 is possibly regulated by COX-2, Survivin, PARP, and Caspase-3 through the activation of histamine receptor H2R or H4R which have been downregulated. It might be possible that this regulation of β-catenin with Survivin may be involved in cell survival by the activation of H1R, H2R, and H4R in EL-4 which are upregulated by MC mediator treatment. No such regulation of Histamine receptors or downstream signaling is seen in L1210 cells, which appear inert to effects of MC mediators. Thus, MC mediators, activate the β-catenin pathway through the histamine receptor downstream signaling, which provides a molecular enlightenment to form a connection between inflammation and cancer.

## Data Availability Statement

All datasets generated for this study are included in the article/Supplementary Material.

## Ethics Statement

The animal study was reviewed and approved by Institutional Animal Ethics Committee (IAEC) JNU, New Delhi (registration no: 19/GO/ReBi/S/99/CPCSEA). Written informed consent was obtained from the owners for the participation of their animals in this study.

## Author Contributions

NP conceived and designed the experiments and contributed reagents, materials, and analysis tools. SP and DM performed the experiments. SP, DM, and NP analyzed the data and wrote the paper.

### Conflict of Interest

The authors declare that the research was conducted in the absence of any commercial or financial relationships that could be construed as a potential conflict of interest.

## Abbreviations

MCs: mast cells
ECM: extracellular matrix
qRT-PCR: quantitative reverse transcriptase polymerase chain reaction
VEGF: vascular epidermal growth factor
G-CSF: granulocyte-colony stimulating factor
TNF: tumor necrosis factor
IL-4: interleukin-4
PBS: phosphate buffered saline
DNP-BSA: 2,4-dinitrophenylated bovine serum albumin
MTT: 3-(4,5-dimethylthiazol-2-yl)-2,5-diphenyltetrazolium bromide
DMSO: dimethyl sulfoxide
FITC: fluorescein isothiocyanate
7AAD: 7-aminoactinomycin D.
