# Supplementary material for: Mast Cells May Differentially Regulate Growth of Lymphoid Neoplasms by Opposite Modulation of Histamine Receptors
Source: Front Oncol. 2019 Nov 21;9:1280. doi: 10.3389/fonc.2019.01280 (PMC6881378; doi:10.3389/fonc.2019.01280)
Supplement: Supplementary file 1 [file Data_Sheet_1.doc]

Mast cells may differentially regulate growth of lymphoid neoplasms by opposite modulation of histamine receptors

# Supplementary Information

**Supplementary Table-1:** Description of various tumor cell lines used

| Cell Line | Disease | Derived From |
| --- | --- | --- |
| YAC-1 | Lymphoma | A/Sn |
| EL-4 | Lymphoma | C57BL/6 |
| L1210 | Lymphocytic Leukemia | DBA subline 212 |

**Supplementary Table-2:** Description of various genes used in the study along with their forward and reverse primers used for their amplification by RT-PCR and their expected product length

| Gene | Forward Primer | Reverse Primer | Product Length |
| --- | --- | --- | --- |
| *Histamine Receptor 1 (HR1)* | 5’-CCTCCGAAGACAAGATGTGTGA-3’ | 5’-AGTGATGCCAGCCAAGTATAGG-3’ | 477bp |
| *Histamine Receptor 2 (HR2)* | 5’-TGCAGGTCAACGAGGTATATGG-3’ | 5’-GCATAACCCAACCATAGGACGA-3’ | 310bp |
| *Histamine Receptor 3 (HR3)* | 5’-AGACTACCTACTGTGTGCCTCC-3’ | 5’-CTGAGGAAGGGTGTGAAGAACT-3’ | 300bp |
| *Histamine Receptor 4 (HR4)* | 5’-GTCATCTTAGCCTTTGTGGTGG-3’ | 5’-GTCCTTTGTGTTCGTGCTGTTC-3’ | 390bp |
| *Survivin* | 5’-CAGCTGTACCTCAAGAACTACC-3’ | 5’-CCCAGCCTTCCAATTCCTTA-3’ | 172bp |
| *Cox-2* | 5’-GTCATTGGTGGAGAGGTGTATC-3’ | 5’-GATGCTCCTGCTTGAGTATGT-3’ | 196bp |
| *GAPDH* | 5’-GTCGGTGTGAACGGATTTGG-3’ | 5’-CTAAGCAGTTGGTGGTGCAG-3’ | 475bp |

**Supplementary Table-3:** RT-PCR gel band intensities of *Survivin, COX-2 and GAPDH* expression in YAC-1, EL4, L1210 and mouse splenocytes treated with MC mediators.

|  | Gene | RPMI | Sensitized MC sup | Resting MC sup | Activated MC sup |
| --- | --- | --- | --- | --- | --- |
| YAC-1 | *COX-2* | 06397.0±07.8 | 06340.1±05.6 | 06450.5±51.7 | 00222.3±05.7 |
|  | *SURVIVIN* | 03445.6±04.8 | 03592.0±06.2 | 03271.5±07.2 | 01236.2±05.5 |
|  | *GAPDH* | 04906.2±22.4 | 04891.1±13.0 | 04591.2±08.3 | 04918.1±30.4 |
| EL4 | *COX-2* | 05783.1±09.3 | 05673.4±01.5 | 05800.1±11.9 | 07533.5±15.9 |
|  | *SURVIVIN* | 03517.4±11.4 | 03312.2±08.1 | 03199.7±06.0 | 05870.1±02.0 |
|  | *GAPDH* | 03996.8±05.5 | 03841.6±06.7 | 03561.7±09.1 | 03561.9±03.2 |
| L1210 | *COX-2* | 06438.6±07.0 | 06598.2±07.6 | 06425.0±08.2 | 06480.6±13.2 |
|  | *SURVIVIN* | 14176.2±17.9 | 14758.8±16.6 | 14729.5±07.5 | 14557.1±09.7 |
|  | *GAPDH* | 11996.8±05.7 | 11841.6±01.8 | 12561.7±06.6 | 11561.9±06.1 |

**Supplementary Table-4:** RT-PCR gel band intensities of *Survivin, COX-2 and GAPDH* expression in YAC-1, EL4, L1210 and mouse splenocytes treated with different concentration of histamine.

|  |  | Control | 0.01µM | 0.1μM | 1.0μM | 10μM |
| --- | --- | --- | --- | --- | --- | --- |
| YAC-1 | *COX-2* | 06784.6±0055 | 06492.1±0046 | 06038.9±0036 | 05290.9±0064 | 05802.5±0053 |
|  | *Survivin* | 04779.2±0051 | 04596.7±0035 | 03893.7±0007 | 02563.1±0036 | 02186.6±0011 |
|  | *GAPDH* | 10774.6±0045 | 10354.2±0028 | 10561.6±0108 | 10826.1±0035 | 10764.2±0023 |
| EL4 | *COX-2* | 04328.2±0021 | 04456.0±0098 | 04564.1±0023 | 06323.4±0005 | 06555.7±0030 |
|  | *Survivin* | 03186.5±0009 | 03169.0±0025 | 04565.5±0044 | 05911.7±0049 | 06010.7±0020 |
|  | *GAPDH* | 04179.0±0024 | 04098.5±0025 | 04108.7±0004 | 04022.8±0014 | 04122.4±0027 |
| L1210 | *COX-2* | 06699.2±0018 | 06561.3±0020 | 06584.1±0004 | 06362.8±0024 | 06555.6±0006 |
|  | *Survivin* | 05166.5±0020 | 05629.0±0045 | 05625.5±0012 | 05249.7±0021 | 05101.1±0007 |
|  | *GAPDH* | 06299.0±0010 | 06580.5±0043 | 06055.7±0024 | 06822.8±0010 | 06198.4±0006 |
| Splenocytes | *COX-2* | 17940.7±0962 | 17815.0±0737 | 18224.0±0426 | 17929.3±0639 | 17670.0±0459 |
|  | *Survivin* | 37932.7±3195 | 38006.3±2654 | 37834.7±3106 | 37227.3±3370 | 37881.0±3469 |
|  | *GAPDH* | 30838.0±0779 | 30415.3±0813 | 30461.7±0776 | 31357.3±0716 | 30766.3±0687 |

**Supplementary Table-5:** Western blot band intensities of Survivin expression in YAC-1, EL4, L1210 treated with MC supernatant.

| Cell line |  | Band intensity (n=3) | Fold change normalized to Actin |
| --- | --- | --- | --- |
| YAC-1 | RPMI | 18503.6 | 1.00 |
|  | Sensitized MC sup | 18547.9 | 0.99 |
|  | Resting MC sup | 16770.1 | 0.89* |
|  | Activated MC sup | 07531.9 | 0.40*** |
| EL4 | RPMI | 17393.3 | 1.00 |
|  | Sensitized MC sup | 17581.0 | 0.99 |
|  | Resting MC sup | 17524.4 | 1.00 |
|  | Activated MC sup | 23826.2 | 1.40*** |
| L1210 | RPMI | 09804.7 | 1.00 |
|  | Sensitized MC sup | 09715.3 | 1.00 |
|  | Resting MC sup | 09733.3 | 1.00 |
|  | Activated MC sup | 09614.1 | 1.01 |

**Supplementary Table-6:** RT-PCR gel band intensities of H1, H2, H3, H4 receptor expression in YAC-1, EL4, L1210 treated with MC mediators

|  | GENE | RPMI control | Sensitized MC sup | Resting MC sup | Activated MC sup |
| --- | --- | --- | --- | --- | --- |
| YAC-1 | H1R | 03673.6±64.5 | 03673.6±04.8 | 03076.3±25.0 | 02585.1±05.6 |
|  | H2R | 02505.3±08.1 | 02588.9±04.7 | 02564.9±08.4 | 02500.1±07.0 |
|  | H3R | 00824.7±07.9 | 00820.2±02.0 | 00865.7±09.1 | 00842.5±09.2 |
|  | H4R | 02005.3±08.4 | 02189.0±12.7 | 02164.9±06.5 | 01800.1±15.3 |
|  | GAPDH | 03782.2±01.7 | 03643.2±05.3 | 03546.0±07.1 | 03599.7±08.5 |
| EL4 | H1R | 01397.0±07.0 | 01340.1±03.6 | 01450.5±04.1 | 02822.3±06.8 |
|  | H2R | 02445.6±05.4 | 02592.0±04.6 | 02271.5±08.5 | 03566.2±03.7 |
|  | H3R | 00906.2±02.9 | 00991.1±01.8 | 00971.1±09.1 | 00918.1±04.1 |
|  | H4R | 00533.6±05.4 | 13327.5±08.7 | 13704.6±38.0 | 16864.6±100.2 |
|  | GAPDH | 03795.1±06.0 | 03704.4±10.9 | 03840.1±02.9 | 03357.2±08.7 |
| L1210 | H1R | 06438.6±05.9 | 06198.3±07.2 | 06425.0±06.6 | 08480.6±04.3 |
|  | H2R | 04176.2±04.6 | 04758.8±03.9 | 04730.0±02.8 | 04557.1±07.0 |
|  | H3R | 01996.7±05.4 | 01841.6±02.5 | 01861.6±03.6 | 01961.9±09.6 |
|  | H4R | 10231.3±04.3 | 10213.7±60.7 | 10199.4±07.3 | 10286.6±08.5 |
|  | GAPDH | 05696.1±05.0 | 05486.4±04.0 | 05256.7±07.0 | 05553.1±04.0 |

**Supplementary Table-7:** RT-PCR gel band intensities of H1, H2, H3, H4 receptor expression in YAC-1, EL4, L1210 treated with histamine at different concentration

| CELL LINE | GENE | Control | 0.01μM | 0.1μM | 1μM | 10μM |
| --- | --- | --- | --- | --- | --- | --- |
|  | H1R | 3673.6±65 | 3673.6±05 | 3076.3±25 | 2585.1±06 | 2145.0±13 |
| YAC-1 | H2R | 2505.3±08 | 2588.9±05 | 2564.9±08 | 2500.1±07 | 2501.0±07 |
|  | H3R | 0824.7±08 | 0820.2±02 | 0865.7±09 | 0842.5±09 | 0893.6±08 |
|  | H4R | 2005.3±08 | 2189.0±13 | 2164.9±07 | 1800.1±15 | 1514.0±21 |
|  | GAPDH | 3782.2±02 | 3643.2±05 | 3546.0±07 | 3599.7±08 | 3604.9±12 |
| EL4 | H1R | 4673.6±09 | 4660.9±09 | 4776.3±07 | 5718.1±09 | 5945.0±23 |
|  | H2R | 1005.3±08 | 1788.9±05 | 1964.9±07 | 2400.1±06 | 3014.0±15 |
|  | H3R | 3124.7±11 | 3120.2±05 | 3265.7±06 | 3142.5±07 | 3193.6±12 |
|  | H4R | 6005.3±08 | 6288.9±09 | 6264.9±21 | 6100.1±03 | 7114.0±12 |
|  | GAPDH | 6782.2±10 | 6643.2±05 | 6546.0±08 | 6499.7±07 | 6504.9±04 |
|  | H1R | 3083.8±10 | 3118.6±16 | 4293.9±10 | 4199.3±07 | 4293.2±04 |
| L1210 | H2R | 2097.3±14 | 2139.5±15 | 2121.8±11 | 2323.5±10 | 2263.5±06 |
|  | H3R | 1299.0±07 | 1290.5±07 | 1335.7±05 | 1282.8±03 | 1288.4±15 |
|  | H4R | 3782.1±04 | 3643.2±06 | 4145.7±08 | 4799.7±07 | 5004.9±07 |
|  | GAPDH | 3102.4±14 | 3173.1±10 | 3164.8±19 | 3164.8±05 | 3114.6±05 |
